# Optimal dosage protocols for mathematical models of synergy of chemo- and immunotherapy

**DOI:** 10.3389/fimmu.2023.1303814

**Published:** 2024-01-19

**Authors:** Urszula Ledzewicz, Heinz Schättler

**Affiliations:** ^1^ Institute of Mathematics, Lodz University of Technology, Lodz, , Poland; ^2^ Department of Mathematics and Statistics, Southern Illinois University Edwardsville, Edwardsville, IL, United States; ^3^ Department of Electrical and Systems Engineering, Washington University, St. Louis, MO, United States

**Keywords:** mathematical model, immunotherapy, chemotherapy, synergy, optimal control

## Abstract

The release of tumor antigens during traditional cancer treatments such as radio- or chemotherapy leads to a stimulation of the immune response which provides synergistic effects these treatments have when combined with immunotherapies. A low-dimensional mathematical model is formulated which, depending on the values of its parameters, encompasses the 3 E’s (elimination, equilibrium, escape) of tumor immune system interactions. For the escape situation, optimal control problems are formulated which aim to revert the process to the equilibrium scenario. Some numerical results are included.

## 1 Introduction

The release of tumor antigen during traditional cancer treatments, such as radio- (1) or chemotherapy (2), can lead to a stimulation of the innate immune response which, in some cases, is able to trigger protective antitumor immunity with possibly long-lasting effects (3, 4). For example, a well-documented abscopal effect of radiation therapy (i.e., the reduction of tumor metastases in areas well outside the field of radiation) is hypothesized of being immune mediated (5–8). These stimulatory effects are the basis for an observed synergy some of these therapies have with immunotherapy, e.g., check-point blockades.

Mathematical models of tumor growth and treatment have a long history going back to the 1980s with research unabatedly continuing (e.g., see (9)). Probably the earliest works on tumor-immune interactions are Stepanova’s paper (10) and (11) by Kuznetsov et al. while mathematical models including immunotherapies are more recent [e.g (12–20)]. Capturing all aspects of tumor-immune interactions in a mathematical model is difficult as the competitive interactions between tumor cells and the immune system are complex, to say the least, and still are the topic of intense medical research. While large-scale, agent-based, PDE, or hybrid models are more precise, they suffer from the inabilities to determine a large number of parameters.

In this paper, we consider a *qualitative*, low-dimensional mathematical model (21). Rather than analyzing the dynamical system for a specific set of parameters, the aim of a qualitative analysis is to understand the totality of all the possibilities the model allows for. Especially for nonlinear models this is an important aspect in pointing out both mathematical limitations and complexities of the underlying dynamics. Motivated by the papers (10, 11, 22, 23) here we consider a model for tumor immune system interactions which in addition to tumor volume and immuno-competent cell densities includes as a third separate variable tumor antigen. This model retains the main aspects of tumor immune system interactions called the three E’s of *cancer immunoediting* (24, 25). These are (i) *elimination*: the immune system is able to completely eradicate the tumor; (ii) *equilibrium*: adaptive immunity is able to maintain cancer in a benign state (tumor dormancy) (26), and (iii) *tumor escape*: tumor growth overcomes or evades the actions of the immune system (27). From a practical (therapeutical) point of view, the first case will never be seen while therapy will not be able to save the patient in the last. Only when it is possible to influence the dynamics (that is, tumor growth) through treatment which will lead to positive changes on a permanent basis, i.e., even after treatment has been stopped, a cure is possible. This requires that the system can be reverted back to the equilibrium situation.

*Optimal control* problems are optimization problems in time in which the full range of possibilities to influence the dynamics of a system, i.e., without *a priori* restrictions on these structures, is considered. This is very different from a “best in class” argument sometimes wrongly also called optimal control in many publications where only a limited number of possibilities is considered, often by mere simulations. Typically in optimal control problems the aim is to transfer the state of a dynamical system from a given initial point into a desired set of terminal states. This is accomplished by minimizing some suitably chosen criterion subject to the dynamics of the system and other constraints that need to be satisfied. Solutions then are functions of time which describe the best actions relative to the chosen criterion. Historically, there has been great success in applying optimal control to engineering problems (moon landing, autopilots on airplanes) and economics (portfolio optimization) while medical applications with its uncertainties in the dynamics—these are generally based on *ad-hoc* modeling premises, not like in physics on first principles—and usually a large parameter uncertainty in the model lack similar success stories. Nevertheless, the scheduling of therapeutic agents over time has all the characteristics of an optimal control problem (28) and there is an increasing bulk of literature in which optimal control is applied to medical problems, e.g., see (19, 29–33). The aim is to minimize some objective related to tumor burden and quality of life of the patient while the underlying system follows the processes of tumor development and treatment interactions. While direct clinical applications are a mere possibility for the future in our opinion, currently the use of optimal control techniques lies more in understanding the dynamics of mathematical models proposed to study medical processes (which contributes to a validation of these models) while solutions to optimal control problems can be helpful in identifying realistic therapy protocols that possibly can be explored in medical trials and practice (28, 31). The contributions of our paper lie in this direction. We formulate an optimal control problem for a qualitative mathematical model of tumor immune system interactions which considers the transfer of the state of the system from a malignant initial condition (corresponding to a tumor escape situation) into a benign state (corresponding to the equilibrium scenario) and show how geometric properties of the dynamics help in formulating and understanding the proper goal of treatment. We discuss the complexities of obtaining optimal controls in this case and highlight some numerical results.

## 2 Methods

A low-dimensional, qualitative model for tumor immune system interactions under chemo- and immunotherapy is formulated as a dynamical system and analyzed mathematically. For the medically relevant scenario of tumor escape (malignant), optimal control problems are formulated whose solutions would revert the system to the equilibrium case (benign).

### 2.1 Mathematical model

We consider the following dynamics (21) for tumor immune system interactions based on classical papers by Stepanova (10) and Kuznetsov et al. (11):


(1) $$\dot{x}=\xi x\left(1-\frac{x}{x_{\infty }}\right)-\theta xy-\alpha xu,$$

(2) $$\dot{y}=a\left(1-bx\right)yz+\gamma -\delta y-\kappa yu+\nu yv,$$

(3) $$\dot{z}=\sigma x+\psi xu-\mu z,$$

State variables are the *tumor volume x*, the *immunocompetent cell density y*, and *tumor antigen z*. The variable *y* is a non-dimensional, order of magnitude quantity which is related to various types of *T*-cells activated during the immune reaction and summarily represents the actions of the immune system. The variables *u* and *v* represent time-varying dose rates *u* = *u*(*t*) at which chemotherapy is given and a time-varying immune boost *v* = *v*(*t*). For simplicity, drug dose rates and concentrations are identified. (It is well-known how to deal with the required changes if standard pharmacokinetic models are included (34). All Greek letters and *a* and *b* are parameters which for the time under consideration are assumed constant. The meaning of variables and parameters is given in **Table 1**.

**Table 1:** Variables and parameters.

variable	interpretation	parameter	interpretation
*x* *x*_∞_	tumor volume tumor carrying capacity	*ξ * *θ*	tumor growth rate tumor-immune interaction
*y*	immunocompetent cell density	*a * *b * *γ * *δ *	tumor antigen stimulated proliferation rate inverse threshold for tumor suppression rate of influx into *y* from primary organs death rate of T-cells
*z*	tumor antigen	*σ * *µ*	intrinsic immunogenicity of the tumor elimination of antigen by the immune system
*u*	concentration of a cytotoxic agent	*α * *κ * *ψ *	chemotherapeutic killing parameter on *x * chemotherapeutic killing parameter on *y * therapy induced boost to immunogenicity
*v*	concentration of an immunotherapeutic agent	*ν*	immune boost

Most of the terms in the equations are standard. Log-linear terms of the Skipper model (35) are used to formulate the damage done to the tumor through the concentrations of the agents and a logistic growth model is used for the tumor volume. This is merely for sake of specificity and analogous results hold qualitatively, for example, for a Gompertzian growth function. Equations 1, 2 follow the classical papers by Stepanova (10) and Kuznetsov (11) and have been taken over with only small changes. Equation 3 extends these earlier 2-dimensional models to include a direct link between tumor antigen *z* and the immuno-competent cell density *y*. This has led to the modified term *a*(1 − *bx*)*yz* used in Equation 2. In (10) this term instead was taken of the form *a*(1 − *bx*)*y* ^2^ with the justification that tumor antigen would be proportional to the tumor volume thus generating the factor *y* ^2^ in the interaction term as tumor antigen is not considered separately in that model. In (2) we have therefore replaced one the factors *y* with *z* restoring a direct link between these two variables. Equation 3 is based on a similar equation in (36) and models the evolution of tumor antigen. It is assumed that the tumor produces antigen *z* at rate *σ* which results in an intrinsic (i.e., not therapy induced) immunogenicity of the tumor. Antigen is cleared by the immune system at rate *µ* which leads to the creation of immune effector cells which generate a stimulating effect onto the proliferation of lymphocytes and thus a positive influx into the compartment determining the immunocompetent cell density *y*. This effect is represented by the term *ayz* in Equation 2. The term *ψxu* models the immuno-stimulatory aspect of therapy assuming that the tumor produces antigen at a dose dependent rate *ψu* with *ψ* modeling the therapy induced immunogenicity of the tumor.

### 2.2 The three E’s of immuno-editing

Depending on the value of the parameters, the dynamical Equations 1–3 properly replicate the full variety of medically realistic scenarios. In order not to be confusing with the *medical notion* of *equilibrium*, we use the terminology *stationary point* for the states which are obtained as solutions when the derivatives in Equations 1–3 are set to zero. There always exists a tumor-free stationary point given by $w_{0}=\;\left(0,\;\frac{\gamma }{\delta },\;0\right)$ . It is stable if *ξδ < θγ* and unstable if *ξδ > θγ*. Intuitively, stability means that solutions of the dynamics which start near *w*_0_ converge to *w*_0_ in time. The relevant term is a difference between products of tumor stimulating parameters (the tumor growth rate *ξ* and natural death rate *δ* of immune cells) and tumor inhibiting parameters (the influx *γ* stimulating the immune system and the effectiveness *θ* of the immune system fighting the tumor). Stationary points with positive tumor volumes *x*_∗_ are zeros of a cubic polynomial *Q* = *Q*(*x*) computed by eliminating *y*_∗_ and *z*_∗_ from the equations $\dot{y}$ = 0 and *ż* = 0. Given the logistic growth model used in Equation 1, only zeros in the range 0< *x*_∗_ < *x*_∞_ are viable solutions for the tumor volume.

The three E’s of immuno-editing correspond to the following scenarios:

#### 2.2.1 Elimination

This situation arises if the tumor-free stationary point is stable and no stationary points with positive tumor volumes exist. All solutions of the dynamics converge to the tumor-free stationary point, i.e., the actions of the immune system are eliminating the tumor. While this is not a relevant scenario medically—in fact, it will never be observed—it nevertheless is part of the complete picture of tumor immune system interactions.

#### 2.2.2 Equilibrium

This situation arises once the tumor-free stationary point becomes unstable and there exists a stationary point with small tumor volume *x*_∗_ (and generally up-regulated *y*_∗_) which is stable. We call this stationary point ‘benign’. There are two different scenarios mathematically which correspond to the medical notion of equilibrium: In the simpler one, the benign stationary point is the only stationary point with positive tumor volume and all trajectories converge to it. In the second case, called the *bi-stable scenario*, there exist three stationary points with positive tumor volumes labelled 0 < *x*_∗_ *_,b_ < x*_∗_ *_,u_ < x*_∗_ *_,m_ < x*_∞_. We call the stationary point with lowest tumor volumes, *x*_∗_ *_,b_*, *benign* and the one with highest tumor volume, *x*_∗_ *_,m_*, *malignant*. This is merely terminology, but it is somewhat justified by the fact that typically the tumor volume *x*_∗_ *_,b_* is small with high *y*_∗_ *_,b_* while *x*_∗_ *_,m_* is high (close to carrying capacity) with low *y*_∗_ *_,m_*. Both the benign and malignant stationary points are stable and we call their regions of attraction (i.e., the set of all initial conditions (*x*_0_ *,y*_0_ *,z*_0_) from which the solution of the dynamics converges to the respective equilibrium point) the benign, respectively malignant regions. The third stationary point *x*_∗_ *_,u_* is unstable and there exists a surface (its 2-dimensional stable manifold) that passes through it which separates the benign from the malignant region, the so-called *stability boundary*. (For 2-dimensional systems the terminology separatrix is common (37)). Depending on where the initial condition for the system lies, as time evolves, for the system without any outside interventions the state will converge either to the benign stationary point— and this also corresponds to the medical equilibrium scenario—or it will converge to the malignant stationary point in which case tumor escape occurs. In a rather precise mathematical sense, the bifurcations (changes in stability) which arise as the values for parameters change characterise the transitions from the medical state of equilibrium to the one of tumor escape.

#### 2.2.3 Escape

In addition to the situation just described, it is also possible that there exists just one viable equilibrium point which, however, has high tumor volume *x*_∗_ and low *y*_∗_, i.e., is malignant. In this case, unless somehow by means outside of the modeling done here a change in the values of the parameters can be achieved, after the termination of any treatment the state of the system will always converge to the malignant equilibrium point and it is not possible to revert to the medical condition of equilibrium. In this case, a cure is elusive.

**Figure 1:**
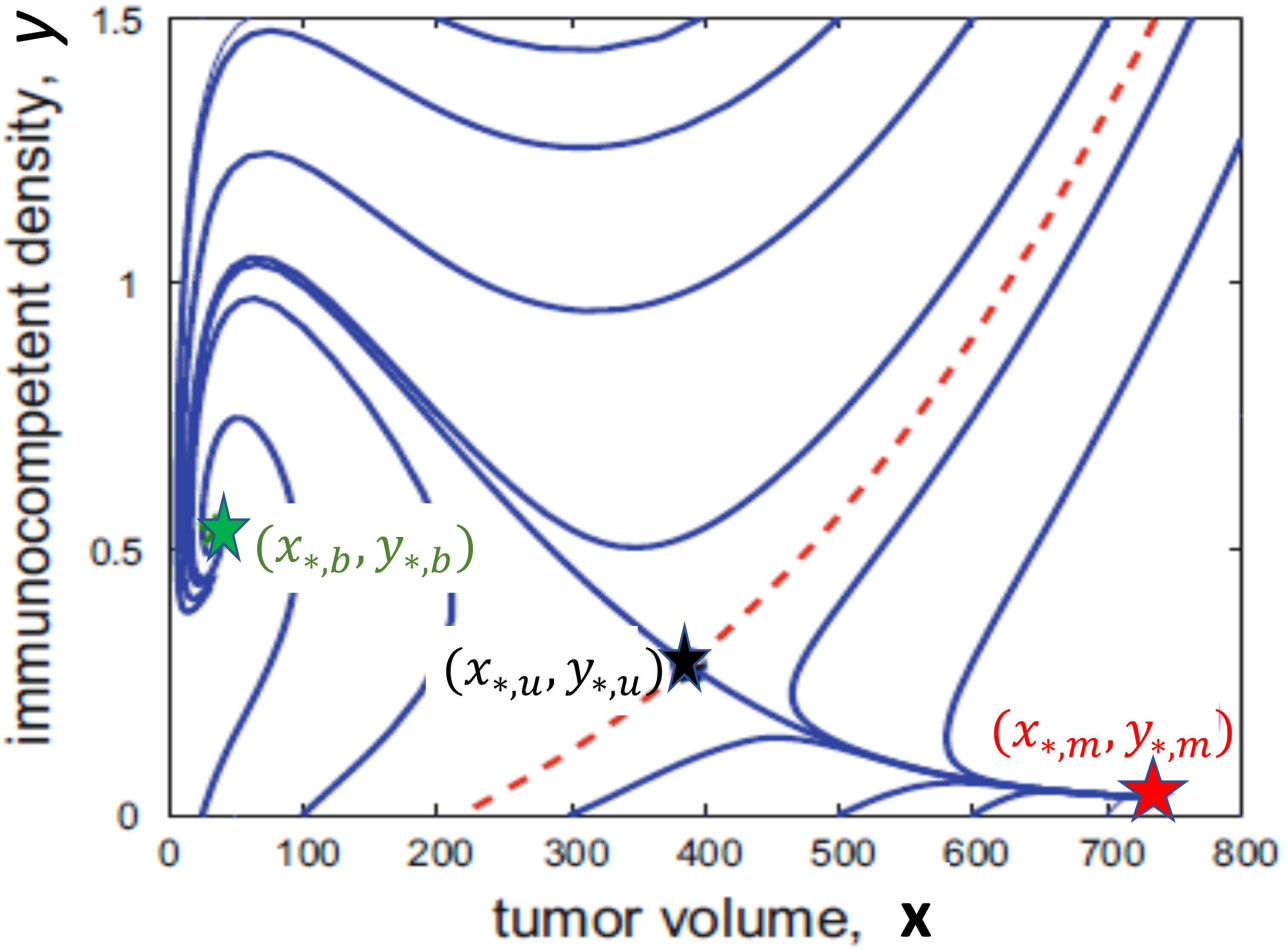
A 2-dimensional illustration of the bi-stable scenario showing the benign stationary point (*x*_∗_ *_,b_,y*_∗_ *_,b_*) in green, the saddle point (*x*_∗_ *_,u_,y*_∗_ *_,u_*) in black and the malignant stationary point (*x*_∗_ *_,m_,y*_∗_ *_,m_*) in red. The dashed red curve is the stability boundary which separates trajectories which converge to the benign stationary point (above the red curve) from those which converge to the malignant stationary point (below the red curve).

We illustrate the role of the stability boundary in the bi-stable scenario through a 2-dimensional representation in **Figure 1**. Formally, we have dropped Equation 3 and replaced *z* in Equation 2 by the equilibrium relation *µz* = *σx*. This thus is not directly related to Equations 1–3, but is merely intended to give an illustration of the underlying geometric scenario. Mathematically it will look the same, but not quite as clearly visible, in a higher-dimensional setting. **Figure 1** faithfully represents the dynamics in the bi-stable case when both a stable benign and malignant stationary point exist. Obviously, whether or not this is the case depends on the parameter values, but it will hold true for an open set, i.e., for a whole range of values. We note that the same feature is present in the original models by Stepanova (10) and Kuznetsov (11), but also in a more recent in spirit similar 2-dimensional model by Bekker et al. (37) (where a slightly simplified dynamics has been used). In that paper the effects of various immunotherapies on shifting the stability boundary are considered for the model. All these low-dimensional mathematical models clearly point out the stability boundary as the defining structure for tumor immune system dynamics. Such a stability boundary only exists in the bistable scenario and for therapy is the only relevant case.

### 2.3 Formulation of treatment as an optimal control problem in the bi-stable scenario

From a practical point of view, only if the current state of the system (initial condition) is malignant the question of treatment arises. Treatment then should aim to move the state into the benign region, possibly in an efficient way or, in other words, one simply wants to minimize the use of agents to limit side-effects. If ‘tumor escape’ can be reversed to the ‘equilibrium’ condition through therapy by moving the state of the system into the benign region, then—but this assumes that parameter values will not change—after therapy is halted convergence to the benign equilibrium point will occur.

Formulating the problem as an optimal control problem can help with singling out reasonable therapy protocols obtained by minimizing some criterion. For the problem considered here, the initial condition is the present state of the system, desired terminal states are in principle all points in the benign region, the dynamics is given by Equations 1–3, and other constraints that need to be considered are related to the side effects of therapy. Optimal controls then give the protocols on how to administer the therapeutic agents in time which are ‘best’ relative to the chosen criterion. Formulating this criterion is a relevant step in this process. If the objective function does not properly represent the overall goal of therapy results may simply not give beneficial suggestions for therapy protocols. When formulating this criterion to be minimized, the following aspects thus must be taken into account:

**(1)** *Minimizing the objective must induce the system to move into the benign region*. For this aim, the geometric shape of the boundary between the benign and malignant regions matters, but there exist many ways to realize such an objective. Here we use a penalty term of the form *V_x_x*(*T*) + *V_y_y*(*T*) + *V_z_z*(*T*) evaluated at the terminal point *w*(*T*) where $\vec{V}=\left(V_{x},\;V_{y},\;V_{z}\right)$ is a suitable vector oriented to point from the benign into the malignant region (as we shall minimize the objective). Generally, one wants to minimize the tumor volume, but it also is the aim to up-regulate the immunocompetent cell density. Thus *V_x_* should be positive while *V_y_* may be allowed to be negative.

**(2)** Side effects of the therapies have not been included in the modeling. Hence these must now be incorporated indirectly by including penalty terms into the objective which limit the overall amounts of drugs given. The total amount of drugs given are measured by the so-called AUC (‘area under the curve’) in pharmacology. This quantity is given by the integral over the dose rate of the drugs: $\int_{0}^{T}u(t)dt$ and $\int_{0}^{T}v(t)dt$ . Alternatively, *a priori* constraints on these amounts could be fixed and then the question would be how to best administer these amounts in time. In the literature often quadratic terms are used for the controls for mathematical expediency, but they have no pharmacological meaning.

**(3)** Mathematically, the existence of a solution needs to guaranteed.

All these considerations led us to formulate the following objective:


(4) $$J=J\left(u,v\right)=V_{x}x\left(T\right)+V_{y}y\left(T\right)+V_{z}z\left(T\right)+\int_{0}^{T}\left(Au\left(t\right)+Bv\left(t\right)+C\right)dt.$$

The objective Equation 4 is a weighted average of ‘good’ and ‘bad’ terms with the components of the vector $\vec{V}$ and the coefficients *A*, *B* and *C* weights. These are *variables of choice* which need to be chosen to strike a balance between the benefit at the terminal time *T* and the overall side effects. As it is standard in engineering approaches, these coefficients should be calibrated to fine-tune the response of the system.

## 3 Results

**Figure 2:**
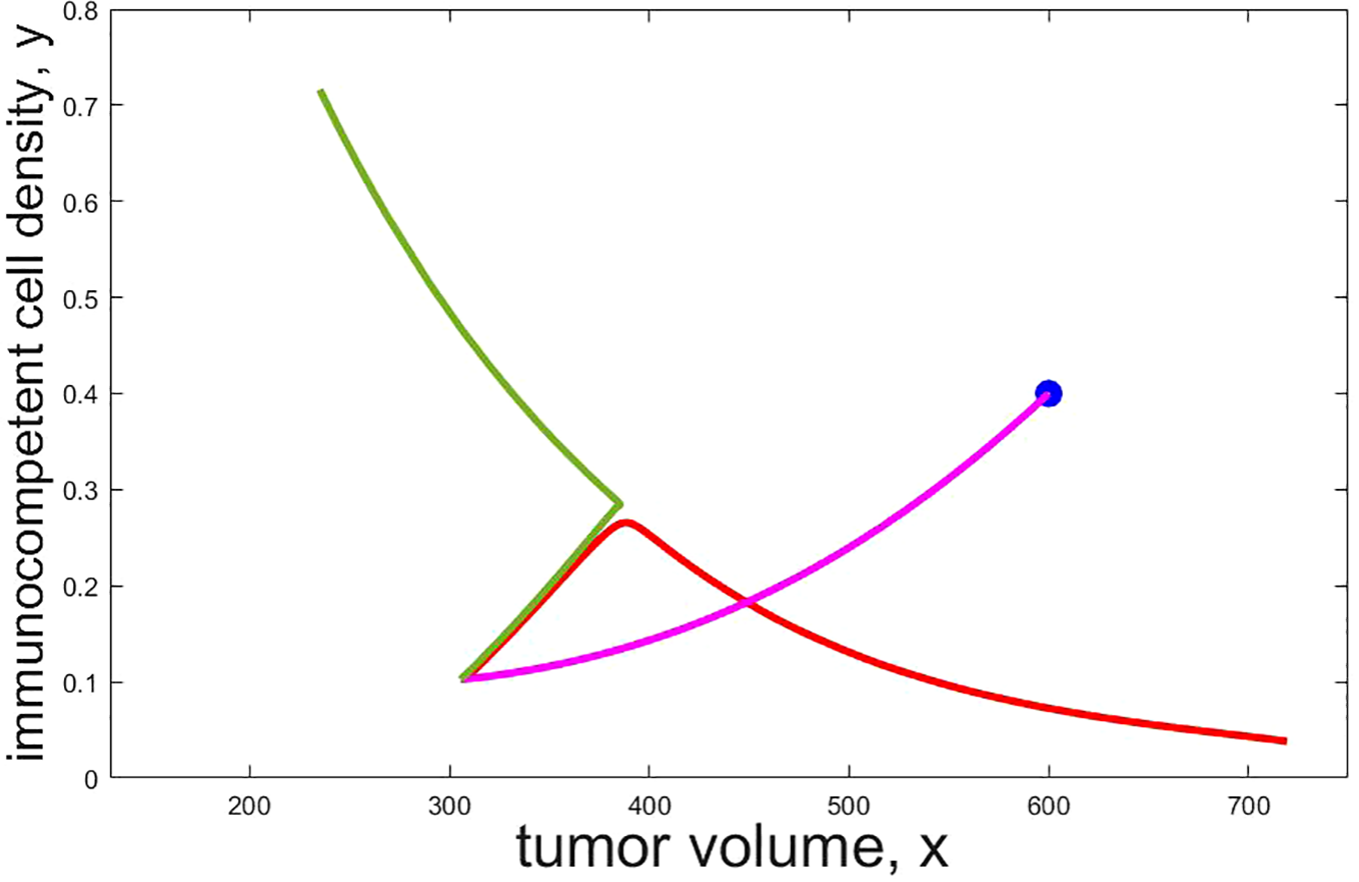
Projections of trajectories which administer both chemo and immunotherapy (*u* ≡ 1 and *v* ≡ 1) from the initial condition (*x*_0_ *,y*_0_ *,z*_0_) = (600,0.40,400) (shown as a blue dot) for time *τ* and then turn off therapy. The initial segment under therapy is shown by a magenta curve, the subsequent trajectories are shown in red (*τ* = 0.72), respectively green (*τ* = 0.73). After therapy is stopped both trajectories still show virtually the same behavior for some time (that is, tumor volume and immuno competent sell density evolve the same way in time) before a separation occurs. Only then the red trajectory converges to the malignant stationary point while the green one converges to the benign one. As this simulation demonstrates, a small change in the administration time of the agents can make a big difference and this may not be recognizable for quite some time from the time history of the states.

Understanding the geometric properties of the stability boundary gives relevant insights into the possible behavior of *ad-hoc* chosen therapy protocols. Given a malignant initial condition, it is a more than reasonable strategy to apply chemo- and/or immunotherapy for some time *τ*, probably chosen by medical guidelines. The time of administration, however, may be crucial. The example in **Figure 2** highlights this importance as it shows that the timing of therapy can make a crucial difference and that what might overall constitute a ‘good’ strategy is anything from obvious. The graphs in the figure show the course of two trajectories in (*x,y*)-space (not their evolution in time) when both chemo- and immunotherapy are applied for time *τ* and then therapy is stopped. The specific numerical values are irrelevant as we merely want to illustrate a general phenomenon which always exists in the bistable scenario, simply caused by the presence of a stability boundary. The initial segment under therapy is shown as the magenta curve, the subsequent trajectories without treatment are shown in red and green, respectively. For the red portion, *τ* = 0.72 and this was not sufficient to move the state of the system into the benign region so that convergence to the malignant stationary point occurs. On the other hand, increasing the time just to *τ* = 0.73 the benign region is reached and subsequently the trajectory converges to the benign stationary point, i.e., the medical condition called equilibrium has been achieved. At the time when therapy is stopped, the states lie close to the stability boundary, but on opposite sides. For a while both trajectories still trace the stability boundary and in each case the tumor volume increases for some time and so does the immunocompetent cell density making it rather impossible to decide whether the course of action was successful or not. Separation of the trajectories only occurs when the state gets close to the unstable saddle point *w*_∗_ *_,u_* where the instability of the saddle becomes dominant and forces the trajectories to converge to one of the stable stationary points. Only at that time the separation between benign or malignant behavior becomes noticeable. If the state is in the benign region, eventually the reaction of the immune system will be strong enough to control the tumor. This clearly points to the importance of transferring the state of the system well into the benign region. Such an objective is easily incorporated in an optimal control framework, but it requires some knowledge about the geometry of the stability boundary.

Considering treatment as an optimal control problem avoids such fallacies as the solutions can be forced to transfer the state well into the benign region. For the problem formulated here a theoretical analysis based on necessary conditions for optimality [e.g., see (38–41)] singles out the following types of therapy protocols as optimal (42, 43): (a) drug administration schedules which alternate between maximum dose-rates and rest-periods, so-called *bang-bang* controls in optimal control (43) and (b) specific intermittent administrations with particular time-varying dosage protocols at reduced rates. These rates are determined by mathematical formulas for what in optimal control theory are called *singular* controls. Such protocols can be ruled as being not optimal for immunotherapy based on a mathematical analysis of the problem with tools of geometric optimal control theory (Legendre-Clesch condition (38, 39, 41).

**Figure 3:**
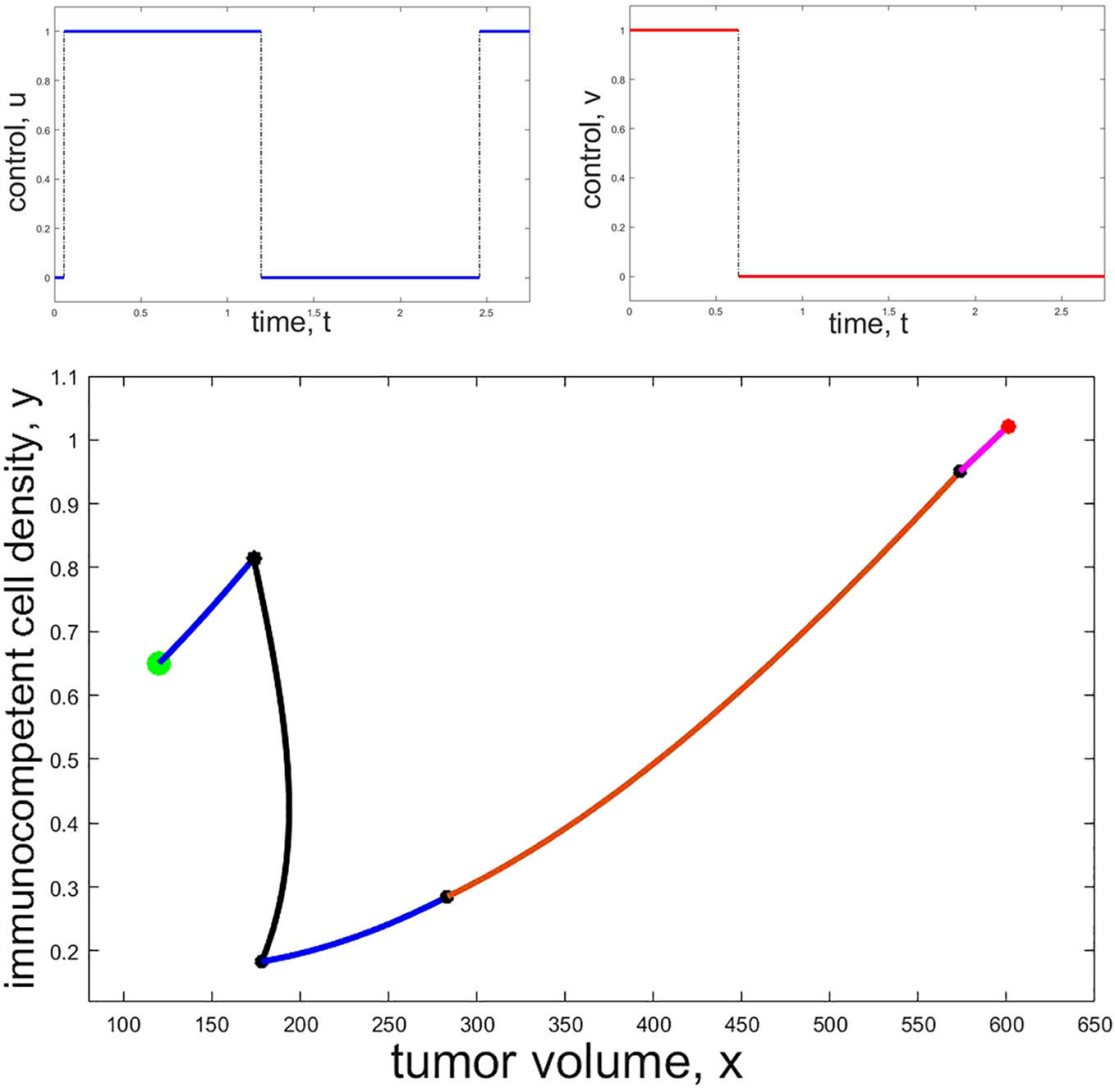
Example of a numerically computed candidate bang-bang trajectory: The top row shows the controls *u* (left, blue) and *v* (right, red) as functions of time while the bottom panel shows the projection of the corresponding trajectory into (*x,y*)-space. The initial point is marked with a red and the terminal point with a green dot. Black dots show the states on the trajectory where a change in the controls occurs. Initially only immunotherapy is given (magenta segment), then both chemo- and immunotherapy are active at the same time (brown segment) while immunotherapy is stopped and only chemotherapy is given along the blue segments. No drugs are administered along the black segment.

**Figure 3** shows a numerically computed candidate bang-bang solution for the problem with a fixed terminal time *T*. The panel shows the controls *u* and *v* and a projection of the corresponding trajectory into (*x,y*)-space. Segments of the curves which correspond to immunotherapy only (*u* = 0 and *v* = 1) are shown as a magenta curve and segments of the curves which correspond to chemotherapy only (*u* = 1 and *v* = 0) are shown as a blue curve. The segment of the curve where both chemo- and immunotherapy are at full dose (*u* = 1 and *v* = 1) is shown as a brown curve and the segment of the curve where none of chemo- or immunotherapy is active (*u* = 0 and *v* = 0) is shown as a black curve. As before, this is merely an episodal illustration.

Unfortunately, and contrary to what seems to be claimed in some publications, there are no “fool-proof” numerical algorithms (not to mention software) which safely compute optimal controls for a mathematical problem of the type considered here. The Hamiltonian function for the control problem is not convex in the control and this precludes the use of simple two-point boundary algorithms. Discretization methods are notoriously unreliable when it comes to locating optimal singular controls often arbitrarily declaring that optimal solutions have been found simply when the value of the objective changes by little. The extremal shown in **Figure 3** was painstakingly computed solving the very sensitive two-point boundary for bang-bang controls using our own code verifying that the result was an extremal. In line with the theoretical results, immunotherapy follows a bang-bang control and here is active at the beginning to be terminated at the second switching time *t*_2_ = 0.630. The more potent chemotherapy is activated almost immediately at the first switching time *t*_1_ = 0.055 and both therapies are on at full dose until the second switching time. The bulk of the reduction in tumor volume occurs during this time-interval. Chemotherapy is terminated at the third switching time *t*_3_ = 1.195 and then only reactivated briefly at the fourth switching time *t*_4_ = 2.4580 close to the end of the therapy horizon. During the long no treatment phase [*t*_3_ *, t*_4_] the tumor volume first increases slightly, but then, as the immunocompetent density increases, starts to decrease again. This behavior is typical for the evolution of a trajectory in a benign region. Therapy then concludes with a brief segment of maximum dose chemotherapy after the prolonged no treatment phase [*t*_3_ *, t*_4_]. While there are no claims made that the parameter values underlying this calculation are medically realistic (they were simply used from various sources to illustrate the structure of possible solutions) and the switching times are merely given to convey some sense of the timing for this particular example, both the controls and trajectories follow reasonable patterns.

The graph in **Figure 3** represents a situation when chemotherapy is significantly more effective than immunotherapy—this was reflected in the numerical values chosen for this particular computation—and is only meant to be representative for such a scenario. Depending on the severity of side effects (represented in the weights of the objective) and the efficacy of the particular agents, different distributions of the administration of the agents will arise. Various examples of locally optimal bang-bang controls for a related optimal control problem are given in (42). Based on our numerical computations, bang-bang controls (administrations of the agents at full dose with rest periods) are the commonly observed optimal protocols for these types of problems.

## 4 Discussion

We formulated a 3-dimensional model for tumor-immune system interactions which in an attempt to more closely model the synergies traditional treatments might have with immunotherapies includes a separate equation for tumor antigen. For a mathematical model of tumor-immune interactions to be credible, it is in our opinion a necessary condition that it encompasses, within its range of parameters, the full spectrum of medically known tumor immune system interactions: elimination, equilibrium and tumor escape. This holds for Equations 1–3. Including tumor antigen as a separate state variable is an attempt to model synergistic effects which traditional treatments (chemotherapy considered here) have when combined with immunotherapies.

We reiterate that the model is qualitatively, not quantitative. The aim is to obtain information about the behavior of the dynamics overall, not about some particular situation. For the latter, a rather precise knowledge of the parameter values is required (which simply lies beyond our possibilities). Our emphasis here was on how understanding the behavior of the dynamics can help in the search for optimal dosage protocols. The optimal control problem allows to explore possible therapy protocols *in silico* suggesting what could be ‘good’ administration protocols relative to some chosen mathematical criterion. Unfortunately, there does not exist off-the-shelf software to solve such optimal control problems reliably and computing optimal controls. As there is great freedom in formulating this objective, there exists the danger of using inadequate criteria for mathematical expediency leading to not only not beneficial but possibly harmful outcomes. Thus we emphasize the need for an a posteriori analysis of the feasibility of the computed therapy protocols. In particular, as side effects of the therapies are only included indirectly while minimizing the objective, the feasibility of the computed strategies from this point of view needs to be checked. It is believed that solutions to these optimal control problems can aid in formulating realistic therapy protocols that can be explored in medical trials and practice.

## Data availability statement

The original contributions presented in the study are included in the article/supplementary material. Further inquiries can be directed to the corresponding author.

## Author contributions

UL: Conceptualization, Supervision, Writing – original draft, Writing – review & editing. HS: Conceptualization, Software, Writing – original draft, Writing – review & editing.
